# Therapeutic Role of Mesenchymal Stem Cell-Derived Extracellular Vesicles in Female Reproductive Diseases

**DOI:** 10.3389/fendo.2021.665645

**Published:** 2021-06-23

**Authors:** Zhiqi Liao, Chang Liu, Lan Wang, Cong Sui, Hanwang Zhang

**Affiliations:** Reproductive Medicine Center, Tongji Hospital, Tongji Medical College, Huazhong University of Science and Technology, Wuhan, China

**Keywords:** mesenchymal stem cells, extracellular vesicle, exosome, reproduction, infertility

## Abstract

Reproductive disorders, including intrauterine adhesion (IUA), premature ovarian insufficiency (POI), and polycystic ovary syndrome (PCOS), are great threats to female reproduction. Recently, mesenchymal stem cells derived–extracellular vesicles (MSC-EVs) have presented their potentials to cure these diseases, not only for the propensity ability they stemmed from the parent cells, but also for the higher biology stability and lower immunogenicity, compared to MSCs. EVs are lipid bilayer complexes, functional as mediators by transferring multiple molecules to recipient cells, such as proteins, microRNAs, lipids, and cytokines. EVs appeared to have a therapeutic effect on the female reproductive disorder, such as repairing injured endometrium, suppressing fibrosis of endometrium, regulating immunity and anti-inflammatory, and repressing apoptosis of granulosa cells (GCs) in ovaries. Although the underlying mechanisms of MSC-EVs have reached a consensus, several theories have been proposed, including promoting angiogenesis, regulating immunity, and reducing oxidate stress levels. In the current study, we summarized the current knowledge of functions of MSC-EVs on IUA, POI, and PCOS. Given the great potentials of MSC-EVs on reproductive health, the critical issues discussed will guide new insights in this rapidly expanding field.

## Introduction

Mesenchymal stem cells (MSCs), a type of adult stem cells, could be harvested from various tissues, including bone marrow, umbilical cord, menstrual blood, endometrial tissue, adipose tissue, etc. (1). Given the capacity of self-renewal and differentiation potentials, emerging researches have regarded MSCs as exciting candidates for cell therapy in regenerative medicine (2, 3). There are lots of pre-clinical and clinical trials confirming the efficacy of MSCs in a variety of diseases, such as cardiovascular disorders, diabetes, neurological diseases, renal fibrosis, and female reproductive disorders (4–8). However, stem cell therapy may raise some negative issues such as transplant rejection, inconvenience of transportation or storage, difficulties of commercialization, and still exhibit safety problems without proper monitoring tests (9, 10). EVs refer to lipid bilayer particles that release from cells into the microenvironment, serving as messengers by trafficking plenty of cargo, such as proteins, microRNAs, lipid and cytokines, and so on (11, 12). In contrast with MSCs, MSC derived extracellular vesicles (MSC-EVs) not only have similar functions with the parent cells, but also exhibit higher biology stability and lower immunogenicity (13, 14).

Female reproductive disorders are great threats to women’s reproductive health and contribute to infertility (15, 16). Although assisted reproductive techniques (ART) have made a great contribution to improving the pregnancy outcomes of infertile couples, women with intrauterine adhesion (IUA) or premature ovarian insufficiency (POI) are still difficult to conceive even with the help of ART (17, 18).

Considering the abovementioned advantages and great potentials in regenerative medicine, MSCs-EVs hold great promise as an alternative therapy for IUA and POI (19, 20). Moreover, recent evidence also indicated that MSC-EVs were functional in improving the ovarian health of women with polycystic ovarian syndrome (PCOS) (21). Accordingly, we draft this review to summarize the therapeutic effects and mechanisms of MSC-EVs on the abovementioned disorders. In this review, the latest studies on the therapeutic effect of MSC-EVs on these diseases are provided and the current limitations and future perspectives of MSC-EVs are discussed as well.

## Methods

For this review, an extensive literature search was performed in PubMed, Embase, and Cochrane libraries. Literature published in English and available up to January 2021 was included.

The following keywords were used for the search, alone or in combination: mesenchymal stem cells, extracellular vesicles, exosomes, female reproductive diseases, intrauterine adhesion, thin endometrium, injured endometrium, endometrial fibrosis, premature ovarian insufficiency, premature ovarian failure, diminished ovarian reserve, polycystic ovary syndrome, follicles, *in vitro* fertilization treatment, angiogenesis, immune regulation, immunosuppressive, collagen remodel, anti-apoptosis, oxidative stress, embryo transfer. Then, the resulting articles were selected by screening titles and reviewing full-text of papers, only articles correlating to the interest topics and its relatives were selected for this review. In addition, we hand-searched references of relevant reviews, and included ongoing studies to locate other potentially eligible materials.

### Extracellular Vesicles From Mesenchymal Stem Cell

**Figure 1:**
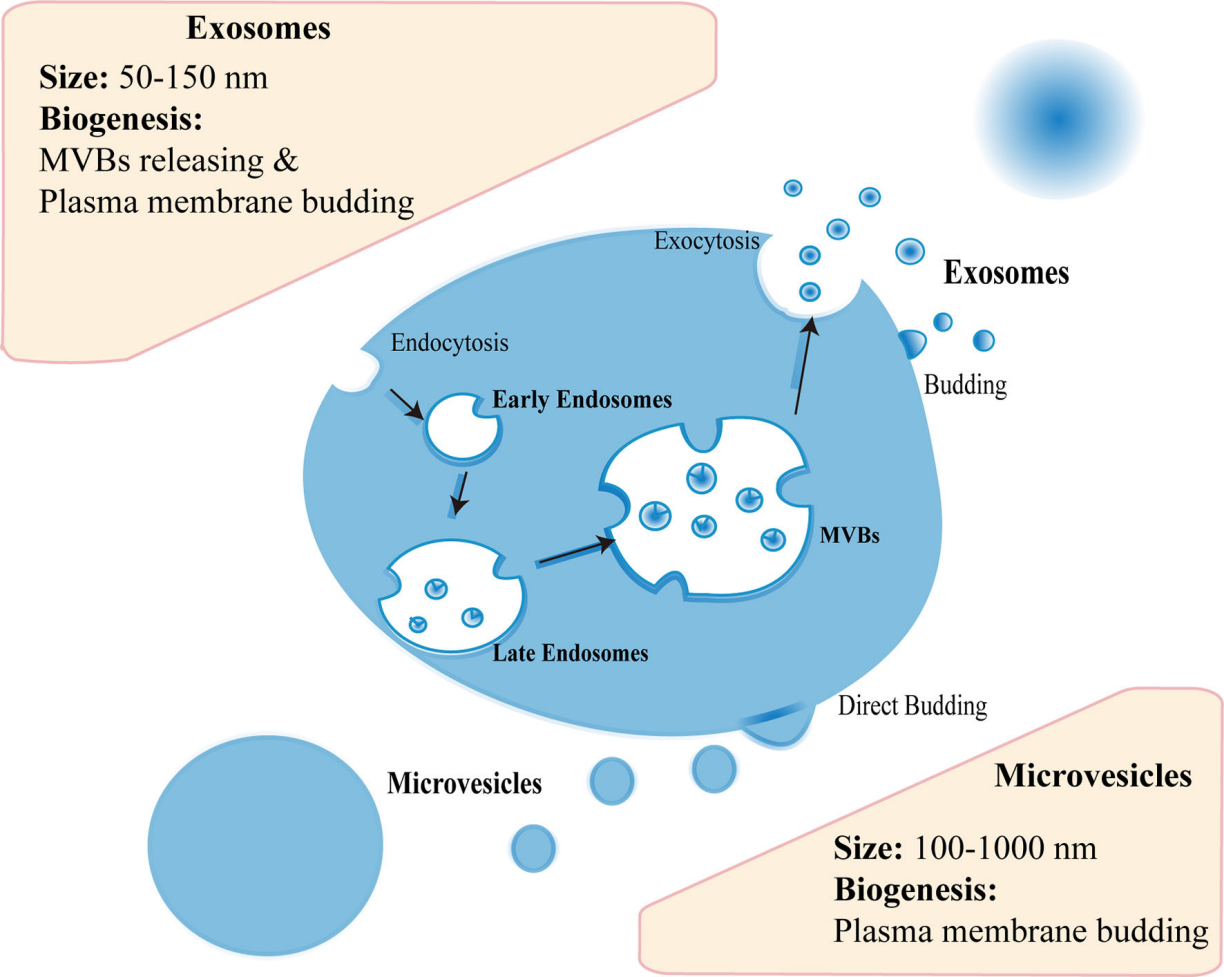
The biogenesis of extracellular vesicles. Exosomes are released upon fusion of MVBs with cell membrane or originate in cell membrane protrusions. Microvesicles bud from the plasma membrane.

Almost all cell types can generate EVs, and MSCs are no exception (22). There are three subtypes of EVs, including exosomes (50–150 nm), microvesicles (MVs) (100–1,000 nm), and apoptotic bodies (500–5,000 nm) (23). The biogenesis mechanism of these subtypes is different to some extent. Generally, the endocytosis and exocytosis account for the biogenesis of most exosomes: initially early endosome can be formed in endocytosis of plasma membrane; with subsequent inward budding of endosomal membrane, late endosome can arise and develop into multivesicular bodies (MVBs) filled with intraluminal vesicles (ILVs); then MVBs release ILVs upon fusion with cell membrane, which mediated by endosomal sorting complexes required for transport (ESCRT)-dependent pathway or ESCRT-independent pathway; finally the vesicles are secreted extracellularly as exosomes (24, 25) (**Figure 1**). As for MVs, the biogenesis is relatively simple, including directly budding from the plasma membrane (26) (**Figure 1**). In fact, evidence showed that exosomes could also originate in cell membrane protrusions (24, 25). Hence, the mechanism of EVs biogenesis needs to be investigated further. As aforementioned, the biogenesis of exosomes and MVs is not alike, their molecular contents or functions are thus dissimilar as well (27). For example, Phinney et al. pointed that the mitochondria could be transferred by MVs, not exosomes, to enhance mitochondrial bioenergetics in macrophages (27). Thus, the functional contents may be different between exosomes and MVs regarding the treatment of female reproductive disorders, which are worthy of exploration.

According to the statement of the International Society for Extracellular Vesicles, ultracentrifugation has been the most commonly used technique to isolate exosomes or MVs, but the resultant EVs are a mixed population owing to the overlaps in the density or size of different EV types (23). As a consequence, it is of importance to characterize individual EV. Conventional means for identifying the characteristics of EVs usually include nanoparticle tracking analysis (NTA) and transmission electron microscope (TEM) observation for morphological information, or western blotting for membrane protein markers (19). Recently, atomic force microscope-infrared (AFM-IR) spectroscopy has been reported to be applied in characterizing individual EV structure and composition, which may provide us a vital tool for deeply understanding subtypes or individual EVs (20).

Generally, the function of EVs is up to their originating cells (13). The therapeutic use of MSCs was reported in female reproductive diseases, so did MSC-EVs (28–30). **Table 1** summarized the major findings of EVs secreted from different categories of MSCs in terms of ameliorating female reproductive disorders. MSC-EVs with different origins exhibited diverse functions, such as repairing injured endometrium, suppressing endometrial fibrosis, regulating immunity, and anti-inflammatory, repressing apoptosis of damaged granulosa cells (GCs), and reducing reactive oxygen species (ROS) level of the ovary (**Table 1**). Although adipose-derived MSC (ADSC)/umbilical cord-derived MSC (UCMSC)/bone marrow MSC (BMSC)–EVs have shown their therapeutic potentials on intrauterine adhesion (IUA) or premature ovarian insufficiency (POI) in small animals (**Table 1**), further studies are required to determine the efficacy and safety of MSC-EVs in primates or even patients.

**Table 1:** Markers and therapeutic effects of reported MSC-derived EVs which contribute to ameliorating female reproductive disorders.

EVs Resource	Species Resources	EVs markers	Major findings	Reference
ADSCs-Exo	Rat/ Human	Alix/ CD9/ CD63/ CD81	1. IUA rat model: Improved endometrial thickness and glands; Decreased fibrotic area; Increased pregnant rate and the number of implanted embryos; Decreased conception time. 2. POI mice model: Increased the number of primordial primary secondary and antral follicles; Increased the level of E2 and AMH; Decreased FSH level; Improved proliferation rate and Inhibited apoptosis of GCs; Increased number of FSHR+/AMH+ GCs and FOXL2+CYP19A1+ GCs. 3. *In vitro* model: Promoted cell growth and inhibited apoptosis of CCs from PCOS patients.	(21, 30, 31)
UCMSCs-Exo	Human	Alix/ CD9/ CD63/ CD81/ Hsp70/ TSG101	1. IUA rat model: Promoted epithelium repair and neovascularization; Improved endometrial thickness and glands; Decreased fibrotic area; Decreased IL-1, IL-6, TNF-α; Increased CD140b, RUNX2, and ER/PR; Increased pregnant rate and implantation sites. 2. TE rat model: Improved proliferation of endometrium; Upregulated VEGF and Bcl-2 level; Decreased caspase-3 level. 3. POI rat/mice model: Decreased the apoptosis and stress of damaged GC; Increased E2 and AMH level; Decreased FSH level; Improved ovarian weight, follicle number, and oocyte retrieved; Reduced conception time; Improved offspring weight; Anti-apoptosis of GCs; Attenuated ROS level. 4. *In vitro* model: Anti-apoptosis; Increased Ki-67 level and follicular count; Increased E2 and AMH; Decreased FSH and ROS level of GCs from POI women.	(29, 32–37)
UCMSCs-MVs	human	CD9 CD63/ TSG101	1. POI mice model: Increased body weight, follicular number and E2 level; Decreased atretic follicles and FSH level; Promoted angiogenesis.	(38)
BMSCs-Exo	Rabbit/ Rat/ Mice	CD9/ CD63/ CD81/ HSP70	1. IUA rabbit model: Increased endometrial glands number; Decreased fibrotic area; Reversed EMT. 2. IUA rat model: Improved endometrial thickness and glands; Decreased fibrotic area. 3. POI rat/mice model: Restore normal estrous cycle; Increased follicular number, E2, and AMH; Decreased FSH and LH; Improved GCs viability.	(39–42)
AMSCs-MVs	equine	/	1. *In vitro* model: Improved endometrial cell proliferation and anti-apoptosis of LPS-treated cells; Decreased TNF-α, IL-6, MMP1, and MMP13 level.	(43)
AMSCs-Exo	human	Alix/ CD9/ CD63/ CD81/ TSG101	1. POI mice model: Anti-apoptosis and elevated proliferation in ovaries; Repressed oxidative stress genes in ovaries; Restore follicular numbers; Increased E2 and AMH level; Decreased FSH level; Promote oogenesis. 2. *In vitro* POI model: Anti-apoptosis of GCs induced by CTX.	(44)
AFMSCs-Exo	Rat/ mice	/	1. POI rat/mice model: Increased AMH; Decreased PTEN and caspase3; Increased estrous cycle; Improved viable offspring and follicular count; Prevented follicular atresia; Anti-apoptosis of damaged GCs	(45, 46)
uMSCs-Exo	rat	**/**	1. IUA rat model: Decreased fibrotic area; Increased MMP-2 and MMP-9 level; Decreased TIMP-1 level; Increased CD31 and VEGF level.	(47)
endMSCs-EVs	Human menstrual blood	CD9/ CD63	1. Embryo maturation: Improved total cell the number of embryos obtained from murine and blastocyst hatching rate. 2. IVF murine model: Improved embryos yield and quality in aged Murine.	(48, 49)

EVs, Extracellular Vesicles; EXO, Exosomes; MVs, Microvesicles; MSCs, Mesenchymal stem cells; ADSCs, Adipose-derived MSCs; UCMSCs, Umbilical cord-derived MSCs; BMSCs, Bone marrow MSCs; AMSCs, Amniotic MSCs; AFMSCs, Amniotic fluid MSCs; uMSCs, uterus derived MSCs; endMSCs, endometrial MSCs; IUA, Intrauterine adhesion; POI, premature ovarian insufficiency; TE, Thin endometrium; E_2_, Estradiol; GCs, Granulosa cells; CCs, Cumulus cells; FSH, Follicle-Stimulating Hormone; LH, Luteinizing hormone; AMH, Anti-Mullerian hormone; CTX, Cyclophosphamide; ROS, Reactive oxygen species; IVF, In vitro fertilization; LPS, Lipopolysaccharides; MVD, Micro-vascular density; HUVECs, Human umbilical vein endothelial cells; IL, Interleukin; TNF-α, Tumor necrosis factor alpha; MMP, Matrix metalloproteinase; TIMP, Tissue inhibitor of metalloproteinase; VEGF, Vascular endothelial growth factor; ER, Estrogen receptor; PR, Progesterone receptor; ECM, Extracellular matrix.

Compared with small animals, primates or human needs a higher dose of EVs. Currently, small-scale production of EVs is a restriction for the clinical application, thus, it is important to find new methods to scale up EV production (50). Previous evidence indicated that MSCs culture parameters were significant for EV production and functions. For example, Patel et al. found that enhanced production of EVs per cell of BMSCs resulted from lower cell seeding density in the culture flasks, and high vascularization bioactivity of EVs was within passage 4 of BMSCs (14). For 3D culture, Watson et al. utilized a hollow-fiber bioreactor to produce bioactive EVs (51). This bioreactor culture model yielded more than 40-fold EVs without serum protein contaminants in comparison with conventional cell culture. Besides, Haraszti et al. also developed a microcarrier-based 3D culture system in combination with tangential flow filtration as a scalable production method for MSC-EVs (52). Moreover, EV yield varies among different MSC cell types (52). With regard to the application method, we are presently witnessing the utilization of MSC-EVs with biomaterials developed rapidly, including collagen scaffold, extracellular matrix mimicking nanofibrous scaffolds, and 3D engineered scaffolds (32, 53, 54). All of them may be very useful for the future use of MSC-EVs in female reproductive diseases. In brief, in terms of clinical application of MSC-EVs, we should take several aspects into consideration, including MSC sources, MSC-EVs production, and usage approaches, which can help us to build a platform of MSC-EVs for clinical application and shorten the time from bench to bedside.

### Therapeutic Effects of MSC-EVs on Female Reproductive Disorders

#### IUA

IUA, also known as Asherman’s syndrome (AS), is characterized by the damage of the endometrial basalis layer and consequent obliteration of endometrium by fibrous tissues (17). Patients with IUA presented with decreased volume of menstrual flow, recurrent pregnancy loss, aberrant placental implantation, and infertility (55). Besides, the endometrium of patients with recurrent IUA is usually very thin, that is, thin endometrium (TE) occurs (56). Multiple factors that interfere with the homeostasis of the uterine environment might be related to this condition, such as artificial abortion, curettage, chronic endometritis, and retained placenta (57). It is acknowledged that endometrial fibrosis is involved in the formation and progression of IUA (58). Initially, the impaired endometrium cannot be normally repaired, which may trigger immune activation and lead to inflammation response along with the over-deposition of extracellular matrix (ECM) protein (collagen and fibronectin) (58–60). As a result, the persistent inflammatory irritation promotes the formation of aberrant avascular fibrotic areas, which may cause tissue hypoxia and sequentially impede endometrial repair (60).

**Figure 2:**
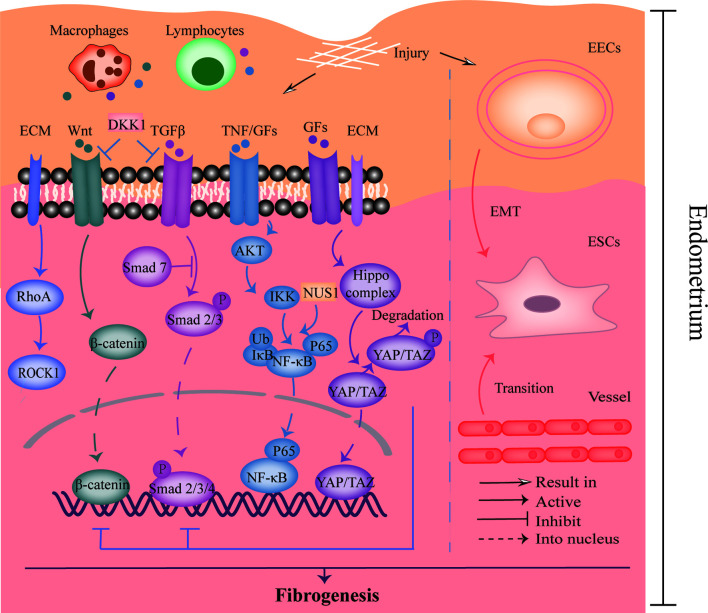
The pathogenesis of IUA. The left part showed the crosstalk of TGF-β, Wnt, NF-κB, Hippo, and RhoA/ROCK signaling pathways, which was relevant to endometrial fibrosis. The right part revealed the process of EMT and endothelial to mesenchymal transition that was involved in fibrogenesis. DKK-1, Dickkopf-1; ECM, Extracellular matrix; GFs, Growth factors; TNF, Tumor necrosis factor; EECs, Endometrial epithelial cells; ESCs, Endometrial stromal cells; EMT, Epithelial-mesenchymal transition.

The pathogenesis of IUA is shown in **Figure 2**. Many studies indicated that the activation of transforming growth factor-β1 (TGF-β1)/Smad3 signaling pathway participated in the occurrence of IUA (61, 62). Moreover, inflammatory factor NF-κB was also identified as a risk factor for IUA (63). Xue et al. observed the expression of TGF-β and connective tissue growth factor-2 (CTGF/CCN2) were positively related to NF-κB pathway activity in the endometrium of IUA patients, and the expression of TGF-β was decreased after inhibiting NF-κB signaling pathway (64). Moreover, it has been reported that the aberrantly activated Wnt/β-catenin pathway was confirmed to stimulate TGF-β-mediated fibrosis and mediate fibrogenesis in the endometrium (65, 66). FOXF2 protein, which activated Wnt/β-catenin pathway and upregulated Collagen Type V Alpha 2 (COL5A2) transcription in the endometrium, was reported to promote fibrogenesis in the IUA as well (67, 68). Therefore, the interaction between many proteins or signaling pathways is of significance to the pathogenesis of IUA.

Bioinformatic analysis revealed an evident cell heterogeneity in the uterus: endothelial cells, stromal cells, fibroblasts, M1 macrophages, mast cells, T cells, and smooth muscle cells (69). Understanding the intercellular interactions and cellular trans-differentiation may enable the pathogenesis of IUA to be elucidated and provide novel treatment targets. For instance, in this study, a heatmap showed the interaction between endothelial cells, especially endothelial cells in endothelial to mesenchymal transition stage, and fibroblasts or stromal cells was high, suggesting the communication might be involved in the progression of IUA. Moreover, the epithelial-to-mesenchymal transition (EMT) also plays an important role in IUA progression (65). A study with the use of IUA rat models revealed the EMT process was promoted by NUS1 protein overexpression *via* regulating AKT/NF-κB pathway, which could be attenuated by microRNA (miR)-466 (70). In contrast, another study showed that miR-1291 promoted endometrial fibrosis through acting ArhGAP29 negatively to upregulate RhoA/ROCK1 pathway that is also relevant to EMT (71). In addition, the Hippo/TAZ signaling pathway was also implicated in regulating EMT process negatively (65). Herein, activation of Hippo pathway would result in the phosphorylated TAZ, consequently inhibiting EMT process (65). Zhu et al. further found that Hippo pathway, which was stimulated by menstrual-blood derived stem cells (MenSCs), could inhibit TGFβ-mediated activation of myofibroblast phenotypes of endometrial stromal cells (ESCs) (72). Taken together, it is essential to treat AS *via* regulating the alternation of cell phenotypes in endometrium that is involved in IUA pathological process.

As shown in **Table 1**, the application of MSC-EVs on AS has been investigated in animal and *in vitro* experiments (30, 32, 43). For example, Yao et al. found that BMSC-EVs promoted endometrial glands, decreased the fibrotic area, and even reversed EMT process in the rat IUA models (39). In this study, injection of BMSCs-EVs significantly declined vimentin (VIM) level and increased the cytokeratin (CK) 19 level. Besides, the expression of TGF-β1, TGF-β1R, and Smad2 was also lower in the treatment group, suggesting BMSC-EVs might repair endometrium by inhibiting TGF-β1/Smad2 signaling pathway (39). Additionally, in Saribas’ work, the injection of uterus-derived MSC_(uMSC)–EVs into the uterine cavity promoted angiogenesis in the IUA rats by increasing the expression of vascular marker CD31 and vascular endothelial growth factor receptor 1 (VEGFR1) (47). Recently, a conference paper reported that UCMSC-EVs promoted rat TE repair by upregulating VEGF, Bcl-2 level and decreasing fibrosis area, suggesting that the regeneration of endometrium could be improved by MSC-EVs (33). Moreover, MSC-EVs may impede IUA progress by inhibiting inflammation. UCMSC-EVs could repress the inflammatory factor interleukin (IL)-1β, IL-6, and tumor necrosis factor (TNF)-α, and increase anti-inflammatory factor IL-10 expression (32). In terms of fertility reservation, the implantation and pregnancy rates were analyzed in this study. Results revealed that UCMSC-EVs could improve these two rates respectively in IUA rats, indicating that UCMSC-EVs might be beneficial to restore infertility of IUA patients (32). Hence, MSC-EVs may serve as a new strategy for treating AS by ameliorating endometrial condition, impeding fibrosis process, promoting angiogenesis, and exerting immunomodulation effect.

However, the application of MSC-EVs in IUA patients is still in its infancy. Several challenges exist in terms of utilizing MSC-EVs in human-beings. First, the mechanisms of MSC-EVs on IUA have not been fully understood yet. Additionally, though the injured endometrium could be repaired by MSC-EVs *in vivo*, such therapeutic effects verified in animal models were in a short period. Whether MSC-EVs were functional in a long-lasting time, has not been explored yet. Furthermore, we could not mimic chronic IUA in animal models, the effect of MSCs on chronic IUA has not been determined. Actually, many patients with IUA have suffered for quite a long time, whether MSC-EVs are efficient in treating those patients demand more investigation. Besides, we only know few information of safety about MSC-EVs used in patients, so the safety of MSCs-EVs is required to study as well (73). Notably, Wu et al. reported clinic-grade human embryonic stem cells (hESCs)–derived immunity- and matrix-regulatory cells (IMRCs) that were verified to cure lung fibrosis, had an efficacy and safety profile in mice and primates (74). Moreover, IMRCs was demonstrated to be superior to UCMSCs as their higher expression of proliferative, immunomodulatory, and anti-fibrotic genes. Hence, it emphasized the safety of IMRCs, however, the therapeutic effects required to be determined in IUA models.

#### POI

**Figure 3:**
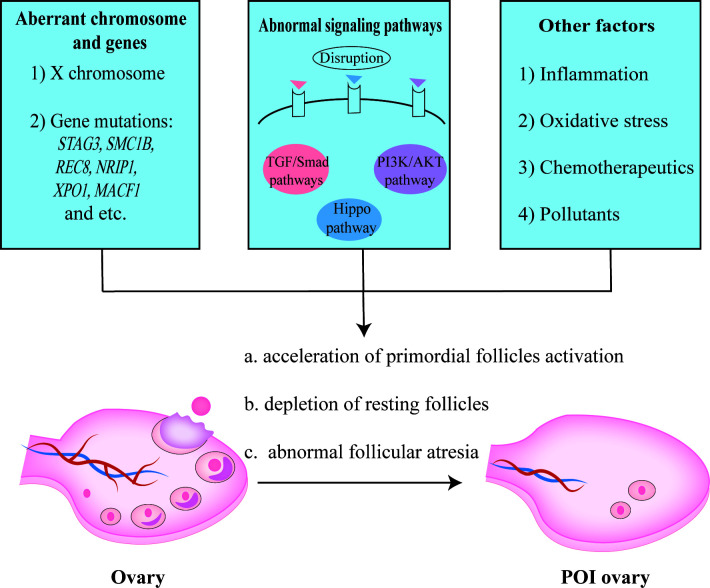
The pathogenesis of POI. Aberrant X chromosome and genes, abnormal signaling pathways, inflammation response, oxidative stress, etc. contributed to the occurrence of POI.

POI is defined by senescence of ovarian function in women less than 40 years old and characterized by amenorrhea or oligomenorrhea for at least 4 months, an elevated FSH level (≥25 IU/L), and fluctuant reduction of estradiol (E2) (75–77). It affects approximately 1% of women under 40 years, which will result in poor infertility outcomes eventually and burden young couples (75, 78). POI might be caused by genetic abnormality, aberrant immunity, chemotherapy or radiotherapy, and environmental pollutants (79, 80). It is believed that primordial follicles cannot be generated and ovarian reservoir is determined at birth, so the ovarian function is easily compromised by accelerated activation of primordial follicles, depletion of ovarian follicles in the resting pool, and abnormal follicular atresia as shown in **Figure 3** (81).

X chromosome and multiple genes are essential for follicle growth and development (82, 83). It has been reported that the inactivation of *STAG3*, *SMC1B*, or *REC8* gene related to meiosis, would result in oocyte arrested with concomitant POI (84, 85). Using whole exome sequencing (WES) analysis, Jaillard et al. proposed new candidate POI genes, including *NRIP1*, *XPO1*, and *MACF1* (86). In fact, it is hard to define a specific causative factor as different genes interplay intricately in the folliculogenesis (81). Apart from gene mutation, abnormal epigenetic modification might also be relevant to an increase of atresia follicles (87–89). Hence, aberrant gene expression or regulation in women can perturb folliculogenesis and bring about follicular atresia, which might lead to the occurrence of POI.

Signaling pathways play critical roles in the follicular development as well, such as PI3K/AKT/mTOR pathway, TGF-β pathway, and Hippo pathway (90, 91). Recently, Grosbois et al. found a synergistical effect of PI3K/AKT and Hippo signaling pathways, wherein primordial follicle recruitment was accelerated and consequently caused rapid depletion of follicles stock (91). Abnormal activation of PI3K pathway could induce the upregulation of AKT/mTOR, while the disruption of Hippo pathway would lead to the de-phosphorylation of YAP/TAZ, both of which resulted in a massive and precocious growth of primordial follicles (91). Whereas repressing PI3K/AKT pathway *via* depleting protein kinase Ck2 contributed to massive follicles atresia in mouse ovaries (92), indicating the significance of PI3K/AKT pathway equilibrium.

Besides, exaggerated autoimmune reaction or inflammatory response also accelerated follicular atresia (75, 93). For example, follicular atresia occurs when GCs continuously exposed to pro-inflammatory cytokines, such as IFN-γ (94). Apart from that, other factors, including unlimited ROS level, some chemotherapeutics, and environmental pollutants, might lead to follicular atresia or depletion as well (80, 81, 95). Notwithstanding, the exact pathogenesis of declined ovarian function remains an enigma, and the relatively complicated pathological mechanism of POI makes it difficult to cure this disease.

Ongoing researches demonstrated that MSC-EVs were able to rescue viability of GCs, suppress ROS level, and restore follicular number in POI animal models (**Table 1**) (31, 34, 38, 96). Recently, AFMSC-EVs were discovered to protect ovarian follicles against gonadotoxic effects of chemotherapy. Herein, miR-10a, a highly enriched miRNA in AFMSC-EVs, promoted resistance to GCs apoptosis or follicular atresia in chemotherapeutics-treated mice (45). After that, Sun et al. also found the UCMSC-EVs reduced cisplatin induced GCs apoptosis *in vitro* (35). And then a study in mice revealed higher level of AKT, P-AKT, VEGF, and IGF in UCMSC-MVs-treated groups compared to non-treated POI group, implying that UCMSC-MVs might induce angiogenesis and activate the PI3K/AKT signaling pathway in ovaries (38). Notably, PTEN, which regulated PI3K/AKT pathway negatively, was downregulated after injected BMSC-EVs or AFMSC-EVs in POI rat models (40, 46), suggesting that MSC-EVs might ameliorate GCs apoptosis in POI *via* PTEN-PI3K pathway. Moreover, MSC-EVs could suppress SIRT families (including SIRT4 or SIRT7) and P53 to reduce cisplatin or cyclophosphamide (CTX)-induced GCs apoptosis (36, 41, 44). Meanwhile, using mRNA and protein assay, Huang et al. found that ADSC-Exo regulated SMAD signaling pathway and rescue GCs from apoptosis (31).

Moreover, MSC-EVs improved offspring outcomes in POI models as well (37). In this study, Liu et al. found that POI mice in UCMSC-EVs transplantation group had higher fertility with less time-to-pregnancy and an increased number of offspring compared to the POI group. Besides, their offspring had nearly similar cognitive behaviors through assessing Y-maze test and novel object recognition task. It was demonstrated that MSC-EVs could improve the fertility of POI mice without adverse effects on the cognitive behavior of their offspring (37).

Similarly, the clinical application of MSC-EVs in POI women is still rudimentary. On one hand, the mechanism of MSC-EVs therapeutic effect on POI is opaque, further researches are needed. On the other hand, the therapeutic effects of MSC-EVs verified on abdominal-injection-constructed animal models, could not totally extrapolate to human patients. Recently, a clinical trial reported a retrograde injection method was used to transplant MSCs based on collagen scaffold (CS) into ovaries of POI patients, suggested CS/MSC-EVs could also be transferred by intra-ovarian injection (97). However, the safety and efficacy of using this method remains to be studied. Additionally, Blazquez et al. noticed the higher blastomere count and hatching rate when murine embryos were exposed to endometrial MSCs (endMSC)-EVs (48, 98). Furthermore, endMSC-EVs were also verified to improve *in vitro* fertilization (IVF) outcome in aged murine model (49). IVF-embryo transfer (IVF-ET) was widely applied in POI patients for assisted conception (99). Perhaps IVF-ET combined with MSC-EVs might be a new method for helping POI patients to conceive, although the safety to offspring needs to be explored.

#### PCOS

PCOS is a common reproductive endocrine disorder characterized by hyperandrogenism, ovulatory dysfunction, polycystic ovarian morphology, obesity, and insulin resistance, which affects about 5–20% of women of reproductive age (98, 100, 101). Hyperandrogenism has been demonstrated as the essence of PCOS (102, 103). Herein, androgen excess was reported to initiate small antral follicle growth and trigger premature luteinization, which inhibited dominate follicle selection and consequently impaired ovulation (102, 103). Anovulatory infertility is a major challenge for women with PCOS, and assisted reproductive techniques are recognized as a last resort to conceive (104). Recently, it was found that *in vitro* maturation (IVM) protocol based on heterologous follicular fluid and GCs supernatant (HFF/GC-IVM protocol) could improve the maturation rate of immature denuded oocytes, fertilization rate, and hatched blastocysts rate for women with PCOS (105). In the meanwhile, ADSC-EVs were noticed to inhibit apoptosis and promote proliferation of cumulus cells (CCs) from PCOS patients, wherein elevated expression of miR-323-3p in exosomes works (21). However, the case of MSC-EVs used in treating PCOS is still few so far.

### Possible Mechanism of Treatment

As described above, though the precise mechanisms of MSC-EVs on female reproductive diseases have not been elucidated yet, several hypotheses have been proposed, including promoting angiogenesis, regulating immunity, reducing oxidate stress level, etc. The functional contents related to above mechanisms in MSC-EVs were summarized in **Table 2**.

**Table 2:** Summary of the functional contents in reported EVs derived from different MSCs.

Contents	Sources	Function	Reference
VEGF	Human ADSC-EV Mice BMSC-EV	Enhanced neovascularization *via* promoting VEGF/VEGFR signaling pathway	(106, 107)
HGF	Mice BMSC-EV	Stabilized endothelial barrier function	(108)
*Jagged1*	Human dental pulp MSC-EVs	Promoted angiogenesis	(109)
*MFG-E8*, *ANGPTL1*, *Thrombopoietin*, *c-kit*, *SCF*	Human ADSC-EV	Promoted angiogenesis	(110)
PDGF, EGF, FGF, NFκB signaling proteins	Human BMSC-EV	Induced angiogenesis	(111)
Wnt4	Human UCMSC-EV	Enhanced angiogenesis through promoting Wnt4/β-Catenin signaling	(112)
Ephrin-B2, Angptl4, PDGFC, Wnt7b, DOK2	Pig ADSC-EV	Induced angiogenesis	(113)
EMMPRIN	CMPC-MSC-Exo	Promoted angiogenesis	(114)
IL-8, miR-21, miR-132, miR-222	MSC-EV	Promoted angiogenesis	(115)
Wnt3a, STAT3	Human BMSC-EV	Promoted angiogenesis and fibroblast proliferation, migration *in vitro*	(116, 117)
miR-125a, miR-30b	Human ADSC-EV	Promoted angiogenesis *via* inhibiting DLL4-Notch signaling pathway	(102, 118)
miR-210	Mice BMSC-EV	Improved angiogenesis, limited fibrosis in ischemic hearts	(119)
miR-130a	Rat BMSC-EV	Promoted angiogenesis	(120)
miR-21	Rat AFMSC-EV	Improved ovarian function	(46)
miR-210	MSC-EV Mice BMSC-EV	Promoting angiogenesis through VEGF pathway, ameliorating inflammation *via* miR-210/*serpine1* axis	(119, 121)
TGF-β	endMSC-EV	Counteracted CD4+ T cells activation,	(122, 123)
	Dog WJMSC-EV	Matrix remodeling	
Let7b	Human UCMSC-EV	Phenotypic conversion of M1 to M2, inhibited pro-fibrotic genes (collagen IVα1, TGF-β1/TGF-βR1)	(124, 125)
CXCL2, CXCL8, CXCL16, DEFA1, HERC5, and IFITM2	MSC-EV	Recruited immune cells to proximity of MSC-EVs	(126)
miR-147	Human UCMSC-EV	Suppressed M1	(127)
miR-182	Mouse BMSC-EV	Induced M2 polarization *via* targeting TLR4.	(128)
miR-223, miR-146b, miR-126, and miR-199a	Human ADSC-EV	Induced M2 polarization	(129)
miR-216a-5p	Human BMSC-EV	Induced M2 polarization	(130)
TSG-6	Human UCMSC-EV	Anti-inflammation	(131)
KGF	Human BMSC-EV	Alleviated inflammation, induced M2 polarization	(132)
IL-10	Human BMSC-EV	Anti-inflammation	(133)
miR-146a-5p, miR-548e-5p	Human AFMSC-EV	Anti-inflammation in human trophoblast cells	(134)
miR-29	MSC-EV	Attenuating renal fibrosis and EMT *via* targeting PI3K/AKT signaling pathway, downregulating TGF-β pathway, or suppressing *snail* expression	(121)
miR-145	MSC-EV	Attenuating EMT *via* inhibiting TGF-β/smad signaling or suppressing ZEB2	(121)
MMP19, ACVR1	Pig ADSC-EV	Matrix remodeling	(113)
MFG-E8	Human BMSC-EV	Attenuated renal fibrosis partly *via* inhibiting RhoA/ROCK pathway	(135)
miR-340	Rat BMSC-EV	Attenuating endometrial fibrosis	(42)
Catalase	Human WJMSC-EV	Decreased ROS level	(136)
miR-320a	Human AMSC-EV	Decreasing ROS level	(44)
miR-17-5p	Human UCMSC-EV	Decreasing ROS level, improved ovarian function	(36)
miR-144-5p	Rat BMSC-EV	Improved ovarian function	(40)
miR-323-3p	Human ADSC-EV	Anti-apoptosis of CCs	(21)
miR-644-5p	Mice BMSC-EV	Anti-apoptosis of GCs	(41)
miR-10a	Mice AFMSC-EV	Anti-apoptosis of GCs, Improved ovarian function	(45)
miR-146a-5p, miR-21-5p	Human UCMSC-EV	Improved ovarian function in aged mice	(135)

VEGF, Vascular endothelial growth factor; HGF, Hepatocyte Growth Factor; MFG-E8, Milk fat globule EGF factor VIII; ANGPTL, Angiopoietin-related protein; SCF, Stem cell factor; PDGF, Platelet derived growth factor; EGF, Epidermal growth factor; FGF, Fibroblast growth factor; NF-κB, Nuclear factor-kappa B; DOK2, Docking protein 2; EMMPRIN, Extracellular matrix metalloproteinase inducer; IL-8, Interleukin-8; TGF-β, Transforming growth factor-β; CXCL, C-X-C motif chemokine ligand; DEFA, Alpha defensin; HERC5, HECT and RCC1 domain protein 5; IFITM2, Interferon inducible transmembrane protein 2; TSG-6, Tumor necrosis factor-stimulated gene-6; KGF, Keratinocyte growth factor; MMP-19, Matrix metalloproteinase-19; ACVR1, Activin receptor type-1; WJMSC, Wharton’s Jelly MSC; CMPC, Cardiomyocyte Progenitor Cells.

#### IUA

It has been well established that MSC-EVs stimulate neovascularization (111, 120, 137, 138). MSC-EVs promoted the formation of tube-like structure formation and spheroid-based sprouting of human umbilical vein endothelial cells (HUVECs) (102, 116, 118). Besides, hemoglobin or CD31+ cells were increased after injecting the mixture of Matrigel and MSC-EVs in mice subcutaneously, indicating the MSC-EVs promoted the formation of functional capillaries (102, 118). Similarly, MSC-EVs increased the expression of VEGF and CD31 in IUA model (29, 47, 139). The contents enclosed in the MSC-EVs might be responsible for such effects. MSC-EVs not only contained multiple pro-angiogenic proteins, such as VEGF and HGF (106). Moreover, several non-coding RNAs (nc-RNAs) enriched in the MSC-EVs, including miR-30b and miR-125a (102, 118). In addition, the following signaling pathways are greatly affected: a) Wnt4/β-catenin pathway (137), b) NF-κB signaling pathway (111), c) VEGF/VEGFR (106, 138), d) PI3K/AKT pathway (120, 140), e) ERK/AKT signaling pathway (114), f) DLL4/Notch signaling pathway (102, 118, 141). Interestingly, these signaling pathways not only were functional in promoting angiogenesis, but also exerted therapeutic effects *via* other mechanisms. For instance, Wnt/β-catenin pathway promoted angiogenesis, and was involved in TGF-β1-mediated fibrosis (67, 142). Therefore, further researches need to characterize the role of pathways in ameliorating IUA by MSC-EVs.

Some researchers proposed that MSC-EVs regulated cell phenotypes, like conferring plasticity of fibroblasts, or inducing mesenchymal-epithelial transition (MET) (39, 143). ADSC-EVs could induce the osteogenic and adipogenic differentiation of human dermal fibroblasts, *via* enhancing the expression of OCT4 and NANOG (143). It presents a new horizon in investigating the mechanism of MSC-EVs in terms of ameliorating IUA.

Besides, emerging studies supported that MSC-EVs had immunomodulatory properties (121, 144, 145). MSC-EVs could guide phenotypic switch of M1 to M2 macrophages *in vivo* and *in vitro* (146). Del Fattore et al. revealed MSC-EVs could promote the proliferation of regulatory T (Treg) cells, which repress immune response through Galectin-1 and PD-L1 (147). In addition, endMSC-EVs and Wharton’s Jelly-derived MSC (WJMSC)-EVs could suppress CD+4 T cell proliferation and activation (122, 123). Moreover, MSC-EVs could modulate immunology *via* regulating cytokines. MSC-EVs were able to downregulate IL-1β, IL-6, and TNF-α levels in LPS-treated endometrial cells (43). Herein, inflammatory pathway, such as NF-κB signaling pathway and JNK/P38 MAPK pathway, could be regulated negatively by MSC-EVs. Contents derived from MSC-EVs, like IL-10, KGF, and TSG-6, could alleviate inflammation as well (131, 133, 148). The role of such factors should be investigated in MSC-EV therapy for IUA (42, 74, 75, 107, 108, 112, 115, 117, 119, 124–126, 129, 131, 131, 149).

#### POI

Apart from promoting angiogenesis (150), MSC-EVs could ameliorate POI *via* reducing oxidative stress (44). Oxidative stress is a phenomenon resulted from accumulation of ROS, which impairs the function and structures of cells and tissues (136, 149). BMSC-EVs could protect cells from toxic effects of peroxide *via* reducing malondialdehyde (MDA) and increasing superoxide dismutase 1 (SOD1) and catalase expression (151). Moreover, MSC-EVs were likely to have a mitochondrial (MIT)-protective effect. The compromised mitochondrial membrane potential (MMP) or ATP level was rescued, and ROS level was reduced significantly after MSC-EVs treatment (151, 152). For this reason, studies about the mechanism of MSC-EVs in ameliorating POI, should not only focus on the effect of anti-apoptosis of GCs under lower levels of ROS, but also further explore the influence of MSC-EVs on MIT dysfunction of GCs or oocytes.

In addition, inflammation response in chemotherapeutic drugs-injured GCs was also inhibited by MSC-EVs, with the decreasing level of IL-6 and IL-1β. The survival rate of GCs was higher in MSC-EV-treated group compared to the model group (153). Collectively, MSC-EVs improve ovarian function mainly inducing angiogenesis, reducing oxidative stress, protecting MIT protective, and regulating inflammation.

#### PCOS

Currently, researches about the therapeutic effect of MSC-EVs on PCOS are still limited, thereby the mechanism of MSC-EVs om improving PCOS has not been elucidated yet. As above mentioned, the dysfunction of follicles is involved in the pathogenesis of PCOS. It has been demonstrated that the communication between oocytes and CCs plays a significant role in the development of follicles (154). Therefore, CCs dysfunction may be related to the decreased oocyte quality and poor pregnancy outcomes of women with PCOS. Zhao et al. found that miR-323-3p transferred by MSC-EVs could ameliorate PCOS *via* promoting growth and inhibiting apoptosis of CCs (21). Previous literature indicated that administration of functional CCs into IVM medium facilitated the oocyte meiosis and embryo development in women with PCOS. Therefore, MSC-EVs, which potentially improve CCs viability, might be a promising treatment for PCOS patients.

As stated above, the functions that MSC-EVs exert depend on the cell types, as the resources of MSC-EVs are heterogeneous. Hence, further studies regarding the therapeutic mechanisms and massive production of MSC-EVs, which may provide reliable evidence supporting clinical applications, are encouraged.

## Conclusions and Future Perspective

MSC-EVs hold great prospects in treating female reproductive diseases, such as IUA, POI, and PCOS. The therapeutic mechanisms included pro-angiogenesis, immunomodulation, anti-fibrosis, and anti-oxidative stress. Although numerous studies confirm the efficacy of MSC-EVs on improving female fertility in *in vitro* and *in vivo* models, such effects may not fully extrapolate to humans. Besides, several questions need to be fully clarified before the application of MSC-EVs in clinic: a) standardized purification and identification protocols for MSC-EVs, b) convenient storage and transportation methods for MSC-EVs, c) determined cargo of large-scale generation of MSC-EVs, d) determined the exact mechanism of MSC-EVs treatment, e) safety issues of MSC-EVs (73). Limited yield is one of the most important problems that restrain the widespread application of MSC-EVs. The production of MSC-EVs might gain benefits from bioreactor culture models (11, 51). For example, hollow-fiber bioreactors or a microcarrier-based 3D culture system are reported to reach industrialized mass production of EVs (51, 52). Herein, a quality-control system should be established to monitor the process of production. Moreover, engineered MSC-EVs may also enhance the efficiency of delivering specific proteins to targeted cells (132, 155). It should be assessed from the effects and safety *via* long-term monitoring.

## Author Contributions

ZL and CS performed data collection and outline design. ZL and CL drafted the manuscript, which was revised by HZ and LW. CL, CS, and HZ contributed equally to this manuscript. All authors contributed to the article and approved the submitted version.

## Funding

This work was supported by National Natural Science Foundation of China (NSFC81901561 and NSFC81771582).

## Conflict of Interest

The authors declare that the research was conducted in the absence of any commercial or financial relationships that could be construed as a potential conflict of interest.
